# Closing gaps: Integrating food safety management systems into the veterinary curriculum a tool to improve food quality and trade

**Published:** 2013

**Authors:** Andrés Cartín-Rojas

**Affiliations:** *Department of Animal Production, Faculty of Agronomy, School of Natural and Exact Sciences, State Distance University, San José, Costa Rica. *

A very important and significant aspect of veterinary public health (VPH) is to enable people to have access and supply to nourishment within an appropriate quality.^1^ In general, the term “quality” refers to the set of properties and characteristics of a product or service that gives it an ability to satisfy the minimum needs of a given consumer.^2^ Thereby, the concept of food quality implies an aliment which has the organoleptic, nutritional, physicochemical and safe characteristics to fit the purpose for human consumption.^3^ Nowadays, maintaining food quality is a key and pivotal element in any industry that manufactures or processes animal by-products obliging them to meet the growing demand for safety from the public and the regulator alike. 

Consequently, preservation of safe food is accompanied by the adoption of methodologies to identify and assess hazards during its manufacturing process,^4^ such as Hazard Analysis and Critical Control Points (HACCP) plans;^5^ or systems that allow the proper risk analysis by implementing promising new procedures used in food industry, for example Failure Modes and Effects Analysis (FMEA).^6^ Thus, the private food sector is being increasingly pressured to ensure that food produced, handled, transported and delivered to the public in compliance with legal and regulatory requirements. In response, during the last two decades a number of private accreditations were established to safeguard food safety (e.g. Global Gap, PAS220, FSSC-22000, ISO 22000:2005, ISO 22005: 2007, etc.). Some of these standards have gained a wide acceptance in several countries during recent years. For example, the International Food Standard (IFS) was created in 2003 which auditing quality management systems in food companies in order to achieve maximum safety. The IFS’s standards are now widely used by the food industry in some European countries such as: Germany, Austria, Belgium, Spain, France, Holland, Poland and Italy.

Currently, livestock sector accounts for nearly half of global agricultural economy. Furthermore, the massive mobilization of livestock commodities in the past two decades has increased the gross domestic product (GDP, a modality for measuring economies and development of nations). Thus, livestock sector in the future will be a growing source of employment and an important tool to alleviate poverty and malnutrition in the developing countries. It is expected with the current production trends, together with a population growth and changes in people’s dietary patterns, the demand for livestock products will double or triple, a process called livestock revolution.^7^ Food risks will increase in parallel, generating those governments should foster in the oncoming decades more efficient mechanisms that allow safeguard consumer’s health. A proper implementation of public policies in food safety requires the integration of all actors along the production chain^8^ under the guidelines of a structured and coherent regulatory framework, in order to enable a transparent, objective and harmonized international trade.^9^ Under the regulatory patterns of World Trade Organization (WTO), through the Agreement on Sanitary and Phytosanitary Measures, the World Organization for Animal Health (OIE) is the normative body responsible for ensuring the control of animal diseases including foodborne zoonoses. Thus, public and private veterinary services are the managers who ensure compliance with these regulatory frameworks. Therefore, it is imperative and crucial that future veterinarians are able to contemplate, audit and even implement these food management quality systems making veterinary profession more pragmatic and adaptable to the new requirements of global livestock markets.

Veterinary services could play an essential role in international markets and trade blocs by certifying the quality of products ensuring that they are free from physical, chemical and microbiological hazards. This will in return improve the confidence of consumers and business partners. This is especially true for any country with an agricultural background which bases its economy on livestock-products exportations. Within this context of global market, Food Safety Management Systems (FSMS) not only harmonizes with the food safety requirements for any food chain, under the binding legislation issued by the OIE. But also, allows that private food industry optimize their resources, maintaining an effective internal and external communication within organizations, and consequently, improve their performance and competitiveness. In addition, FSMS also allows fulfilling and effectively incorporating into the private industrial sector, of international regulatory standards and codes issued by the Codex Alimentarius Commission (CAC) to ensure a seamless coverage of food safety along the manufacturing and value chain.

Therefore, veterinarians professionals should not only assimilate, but promote the implementation of new FSMS methodologies in order to integrate mechanisms of standardization in the manufacturing processes,^10^ allowing that FSMS to be established, operated and updated within the framework by implementing a clear epidemiological and preventive control of foodborne diseases, while amalgamate mechanisms to actively participate and differentiate schemes to increase consumer confidence, improve food security, reconciling food safety and quality control, and allow that food producers, retail and distribution systems will be based on international standards.
